# Genetic diversity and distribution of *Mycobacterium tuberculosis* genotypes in Limpopo, South Africa

**DOI:** 10.1186/s12879-017-2881-z

**Published:** 2017-12-12

**Authors:** N. T. C. Maguga-Phasha, N. S. Munyai, F. Mashinya, M. E. Makgatho, E. F. Mbajiorgu

**Affiliations:** 1 Department of Pathology and Medical Sciences, University of Limpopo, Private Bag X1107, Sovenga, Mankweng 0727 South Africa; 2 School of Anatomical Sciences Faculty of Health Sciences University of the Witwatersrand, 7 York Road, Wits Medical School, Parktown, Johannesburg, 2193 South Africa

**Keywords:** Mycobacterium tuberculosis, Spoligotyping, Genetic diversity

## Abstract

**Background:**

Tuberculosis remains a major health problem and knowledge of the diversity of *Mycobacterium tuberculosis* strains in specific geographical regions can contribute to the control of the disease. This study describes the genetic profile of *M. tuberculosis* in five districts of Limpopo Province.

**Methods:**

A total 487 isolates were collected from the National Health Laboratory Services from all regions/districts of Limpopo Province. Only 215 isolates were confirmed to be *M. tuberculosis* by Bactec Mycobacterium Growth Indicator Tube 960® and Rhodamine-Auramine staining. Isolates were subcultured on Löwenstein-Jensen medium agar slants to validate purity. They were spoligotyped and data analysed using the international spoligotyping database 4 (SpolDB4).

**Results:**

Of the 215 isolates, 134 (62.3%) were genotyped into 21 genotype families while 81 (37.7%) were orphans. The 81 orphans were further subjected to resolution employing SpolDB3/RIM. Overall, the study revealed a high diversity of strains of 32 predominantly the non-Beijing lineages: the LAM- LAM3 (9.8%), LAM9 (4.7%) and LAM11- ZWE (3.3%), the T-T1(15.0%), T2 (0.9%), T2-T3 (1.4%), the CAS-CAS1-Delhi 5 (1.9%) and CAS1-KILI (1.4%) the MANU2 (1.4%), U (0.5%), X-X1(1.4%), X3 (1.9%), S (9.8%), CAS (1.4%), LAM7(0.9%), T3(0.5%), LAM8(4.7%), T4(1.4%), X2(0.4%), AI5(1.9%), LAM1(0.5%), FAMILY33 (1.9%), EAI4(1.4%), *M. microti* (1.9%). The Beijing and Beijing-like families were (14.9%) and (0.9%), respectively. A total of 28(13%) clusters and 77(36%) unique cases were identified. Beijing strain (SIT 1) formed the biggest cluster constituting 14%, followed by LAM3 (SIT 33), T1 (SIT 53) and LAM4 (SIT 811) with 7%, 5.1% and 2.8%, respectively. The Beijing family was the only genotype found in all the five districts and was predominant in Mopani (18.8%), Sekhukhune (23.7%) and Vhembe (23.3%). Dominant genotypes in Capricorn and Waterberg were LAM3 (11.9%) and T1 (13.3%), respectively.

**Conclusion:**

A wide diversity of lineages was demonstrated at district level. A high number of clusters per district provided evidence of on-going transmission in this Province.

## Background

Tuberculosis (TB) remains one of the major health challenges worldwide, despite the availability of a vaccine and treatment options [1]. South Africa is disproportionately affected by TB, with an estimated incidence of 940/100000 persons per year, probably driven by the high HIV frequency in South Africa [2, 3]. The transmission dynamics of MTB lineages may depend on whether they are clustered to indicate an ongoing transmission or unique for reactivated TB [4]. The discovery of polymorphic DNA in *M. tuberculosis* strain has led to molecular typing of M. tuberculosis strains using techniques such as IS6110 restriction fragment length polymorphism (RFLP), spoligotyping and mycobacterial interspersed repetitive units of variable-number tandem repeats (MIRU-VNTR) [5, 6]. Spoligotyping is a rapid reliable and cost-effective method based on analysis of polymorphism in the direct repeat (DR) chromosomal region with identical 36-bp DRs alternating with 35 to 41 bp unique spacers specific for studying the genetic diversity of M. tuberculosis complex members [7]. Spoligotyping has been used in molecular epidemiological studies conducted in South Africa and other countries [8–12]. Nine superfamilies or clades of M. tuberculosis complex have been identified, namely, *M. africanum*, Beijing, *M. bovis*, East African- Indian (EAI), central Asian (CAS), T group of families, Haarlem, X and Latin American Mediterranean (LAM) family [13]. Multiple mycobacterial lineages have been reported in several provinces of South Africa comprising of the Beijing and non-Beijing genotypes [8, 14–16]. However, the studies on South African genetic diversity of M. tuberculosis have been pitched at provincial levels, and most researchers have not paid any attention to such diversity within the provinces except for the study in three areas of the Free State Province [17]. Furthermore, Limpopo Province of South Africa shares its borders with Zimbabwe to the north, Mozambique to the east and Botswana to the west. Genotyping and continuous epidemiological monitoring of circulating strains of *M. tuberculosis* is crucial for TB control program and tracing of transmission. The province is comprised of rural and urban settings and has mining companies in rural areas and cities/towns. There remains possibilities or chances of divers M. tuberculosis complex strains due to increased cross migration from one area to another. A study by Stavrum et al. (2009) on diversity of MTB genotypes targeted 8 provinces in South Africa excluding the Limpopo Province [15]. One study by Sekati, Molepo and Nchabeleng (2015) on molecular characterisation of MTB strains tested only 5 isolates from the Limpopo Province [9]. Therefore, the purpose of this study was to determine the *M. tuberculosis* genotypes, their distribution and diversity around the districts of the Limpopo Province.

## Methods

### Sample size, collection and culturing

**Fig. 1:**
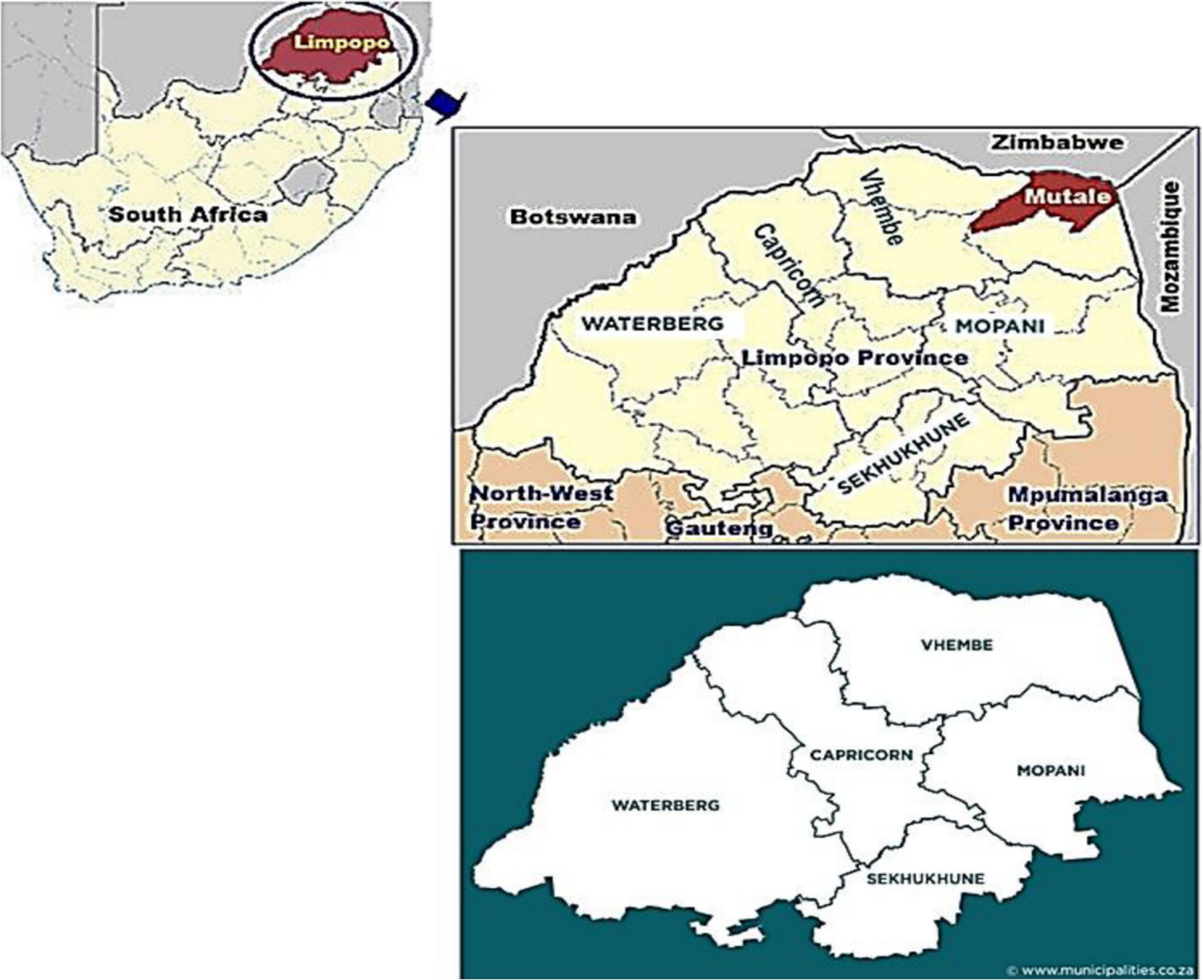
Geographic location of Limpopo Province, its Districts and neighboring countries. Adapted from Khosa et al.,2013 and https://municipalities.co.za/provinces/view/5/limpopo, modified by Mbajiorgu EF

This was a cross-sectional study conducted between November 2012 and November 2013 and hence the sample size was calculated using the formular (Z_1-α/2_)^2^ P(1-P)/d^2^, with d of 0.05 according to Charan and Biswas (2013) [18]. With a proportion of 11.4% TB cases reported by NHLS during the period of the study in Limpopo Province, the required minimum TB positive sample size was 170 with an attrition of 10%. The samples were collected from archived isolates confirmed to be positive for tuberculosis (TB) from the National Laboratory Services (NHLS) in Polokwane. The NHLS is a referral laboratory for regional TB hospitals and clinics in Limpopo Province which provides laboratory and related public health services to more than 80% of the population through a National network of laboratories. Fig. 1 shows the map of South Africa and the location of Limpopo Province and its districts or regions where samples were collected [19, 20].

Convenient sampling was used and data were extracted from NHLS, a major referral facility in the Limpopo Province, including demographic, data were collected from data registry at NHLS in Polokwane hospital covering age range of 1 year to 65 years and above consisting of 254 (52%) and 233 (48%) males and females, respectively. A total of 215 M. tuberculosis isolates identified by a positive rhodamine-auramine stain and a positive Mycobacterium Growth Indicator Tube 960 (MGIT) [NHLS Polokwane] were included in the study. In order to obtain a pure culture of M. tuberculosis strains, a loopful of inoculum from the MGIT tube was sub-cultured onto Löwenstein–Jensen agar slants (Diagnostic Media Products, Johannesburg, South Africa). The L-J cultures were incubated at 37 °C for up to 6 weeks or until there was visible confluent growth.

### Deoxyribonucleic acid (DNA) extraction and spoligotyping

The extraction of DNA from *M. tuberculosis* culture isolates was carried out by suspending a loopful of bacterial colony collected from LJ medium agar slants in 50 μl of distilled water in a screw capped micro centrifuge tube. The bacterial suspension was boiled for 20 min and then centrifuged for 5 min at 14000 g to sediment cell debris. The supernatant containing genomic DNA was recovered and stored at -20 °C until further testing. Spoligotyping was carried out using the commercially available from Ocimum Biosolutions, India, by the standard method described before.7 The direct repeat region (DR) of genomic DNA was amplified using the primers DRa (biotinylated at the 5′ end) and DRb. Chromosomal DNA of M. tuberculosis strain H37Rv and *M. bovis* BCG P3 were included as positive controls. The amplified DNA was subsequently hybridized to a set of 43 oligonucleotide probes by reverse line blotting. The presence of spacers was visualized on film as black squares after incubation with streptavidin- peroxidase and detected with ECL (Enhanced Chemoluminescence System Detection Liquid) (Isogen Life Science).

### Statistical analysis

The data were analyzed using Statistical Package for Social Sciences (SPSS) version 23. The Chi-square test was used to calculate the frequencies of lineages in each district. The results obtained from spoligotyping were entered in a binary format as excel spreadsheets (Microsoft). Spoligotyping patterns of the isolates were matched to the patterns in the Spoligotyping international database (SpolDB4) to identify the lineages. The strains with Spoligotype similar to any pattern of strain already existing in the database were labelled with already deefined ‘shared type’ number. Isolates with no matching pattern in SpolDB4 were regarded as orphans. Isolates with same genetic pattern were regarded as a ‘cluster’, indicating recent transmission of M. tuberculosis, while isolates with different genetic pattern were regarded as ‘unique’, arising from distantly acquired or reactivation of TB infection.

## Results

### Genetic diversity by spoligotyping

**Fig. 2:**
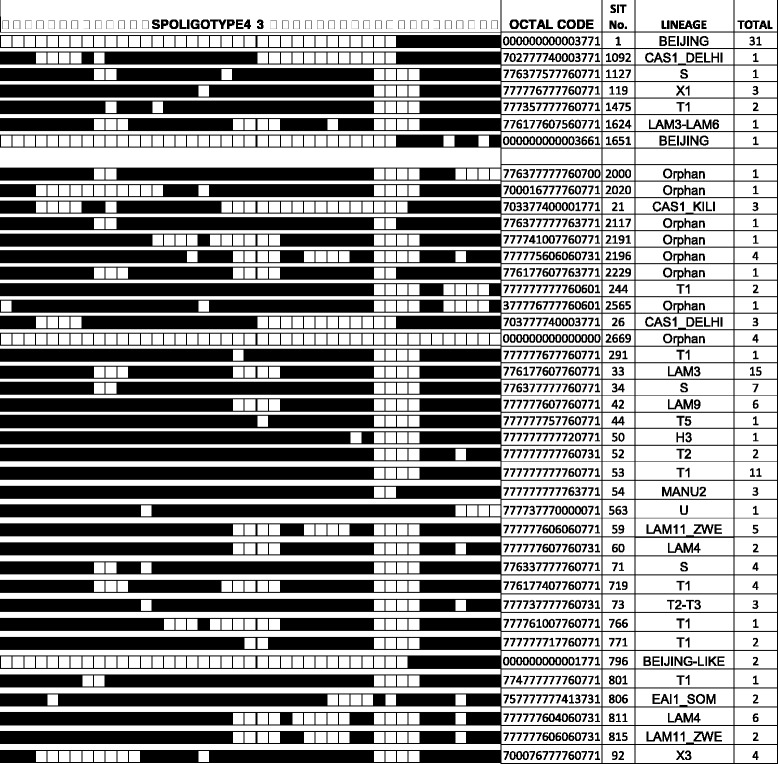
Representation of the Spoligo pattern of different genotypes of *M. tuberculosis* The dark and white boxes indicate the presence and absence hybridization, respectively. Abbreviations of Genotypes as examples: CAS=Central Asian; LAM = Latin American-Mediterranean; H=Haarlem; U=Unknown patterns.

In this study, a total of 21 genotype families were identified. Of the 215 *M. tuberculosis* isolates, 134 isolates (62.3%) had their genotype families identified in the SpolDB4. The remaining 81 (37.7%) had no matching pattern in the SpolDB4 and were termed orphans. Of the 81 orphans 78 were further resolved employing SpolDB3/RIM resulting in 23 LAM, 3 CAS, 4 T, 1 X2, 9 EAI, 4 FAMILY33, 4 *M. microti* and additional 9 S, 4 X1, 9 T, 2 H3 and 2 EAI 1 isolates. The overall Spoligotyping resulted in 28 (13%) clusters and 77(36%) unique cases. The families were assigned to previously described shared types: Beijing (two types of Beijing that differ by octal codes) (SIT 1 and SIT 1092), Beijing-like (SIT 1127) and non-Beijing consisting of the following lineages and sub-lineages: CAS1-KILI (SIT 119), CAS1_DELHI (SIT 1475), CAS1-KILI (SIT 1624), H3 (SIT 50), LAM3 (SIT 33), LAM3- LAM6 (SIT 1624), LAM4 (SIT 60 & 811), LAM9 (SIT 42), LAM11-ZWE (SIT 59 & 815), T1 (SIT 53, 244, 719, 766, 771, 801 & 1471), T2 (SIT 52), T2-T3 (SIT 73), T5 (SIT 44), X1 (SIT 119), X3 (SIT 92), EAI1-SOM (SIT 806), MANU2 (SIT 54), S (SIT 71 & 1127), U (SIT 563), Beijing strain (SIT 1) formed the biggest cluster constituting 14%, followed by LAM3 (SIT 33), T1 (SIT 53) and LAM4 (SIT 811) with 7%, 5.1% and 2.8%, respectively. The results of spoligotyping analysis and a sample Spoligo pattern are presented in Table 1 and Fig. 2, respectively.

**Table 1:** The Distribution of Spoligotype according to districts in Limpopo Province, South Africa

Spoligotype family	Total isolates *N* = 215 (%)	DISTRICTS				
		Capricorn *N* = 101 (%)	Mopani *N* = 16 (%)	Sekhukhune *N* = 38 (%)	Vhembe *N* = 30 (%)	Waterberg *N* = 30 (%)
Beijing	32 (14.9)	10 (9.9)	3(18.8)	9(23.7)	7(23.3)	3(10.0)
CAS1_DELHI	4 (1.9)	1 (1.0)	0(0.0)	0(0.0)	1(3.3)	2 (6.7)
S	21(9.8)	13(6.0)	1(0.5)	3(1.4)	0(0.0)	4(1.8)
X1	7(3.2)	6(5.9)	0(0.0)	1(0.5)	0(0.0)	0(0.0)
T1	33 (15.0)	15(7.0)	1(0.47)	7(3.2)	5(2.3)	5(2.3)
LAM3-LAM6	1(0.5)	0(0.0)	1(6.3)	0(0.0)	0(0.0)	0(0.0)
CAS1_KILI	3(1.4)	1(1.0)	1(6.3)	0(0.0)	0(0.0)	1(3.3)
LAM3	21(9.8)	16(7.4)	0(0.0)	2(0.9)	2(0.9)	1(0.5)
LAM9	10(4.7)	4(1.9)	0(0.0)	2(0.9)	0(0.0)	4(1.8)
T5	1(0.5)	1(1.0)	0(0.0)	0(0.0)	0(0.0)	0(0.0)
H3	31.4()	2(0.9)	0(0.0)	1(0.5)	0(0.0)	0(0.0)
T2	2(0.9)	0(0.0)	0(0.0)	1(2.6)	1(3.3)	0(0.0)
MANU2	3(1.4)	2(2.0)	0(0.0)	0(0.0)	1(3.3)	0(0.0)
U	1(0.5)	0(0.0)	1(6.3)	0(0.0)	0(0.0)	0(0.0)
LAM11_ZWE	7(3.3)	3(3.0)	0(0.0)	0(0.0)	4(13.3)	0(0.0)
LAM4	8(3.7)	1(1.0)	2(12.5)	0(0.0)	3(10.0)	2(6.7)
T2-T3	3(1.4)	2(2.0)	1(6.3)	0(0.0)	0(0.0)	0(0.0)
BEIJING-LIKE	2(0.9)	1(1.0)	0(0.0)	1(2.6)	0(0.0)	0(0.0)
EAI1_SOM	4(1.9)	0(0.0)	0(0.0)	2(0.9)	1(0.5)	1(0.5)
X3	4(1.9)	1(1.0)	0(0.0)	2(5.3)	0(0.0)	1(3.3)
CAS	3(1.4)	2(0.9)	0(0.0)	0(0.0)	1(0.5)	0(0.0)
LAM7	2(0.9)	1(0.5)	0(0.0)	0(0.0)	0(0.0)	1(0.5)
T3	1(0.5)	1(0.5)	0(0.0)	0(0.0)	0(0.0)	0(0.0)
LAM8	10(4.7)	3(1.4)	3(1.4)	3(1.4)	1(1.4)	0(0.0)
T4	3(1.4)	3(1.4)	0(0.0)	0(0.0)	0(0.0)	0(0.0)
X2	1(0,5)	1(0.5)	0(0.5)	0(0.5)	0(0.0)	0(0.0)
EAI5	4(1.9)	2(0.9)	0(0.0)	0(0.0)	1(0.5)	1(0.5)
LAM1	1((0.5)	1(0.5)	0(0.0)	0(0.0)	0(0.0)	0(0.0)
FAMILY 33	4(1.9)	1(0.5)	2(0.9)	1(0.5)	0(0.0)	0(0.0)
EAI4	3(1.4)	1(0.5)	2(0.9)	0(0.0)	0(0.0)	0(0.0)
*M. microti*	4(1.9)	2(0.9)	0(0.0)	0(0.0)	1(0.5)	1(0.5)

*N*number of isolates, Data is presented as Number (percentage)

### Geographical distribution of Spoligotypes

**Fig. 3:**
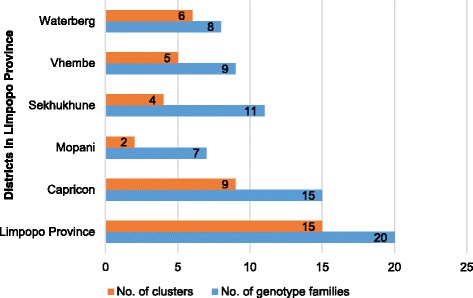
The number of genotype families and clusters in each district

The geographical distribution of *M. tuberculosis* Spoligotypes is shown in Table 1. A comparison of lineages among the five districts shows that the Beijing family was the only genotype found in all the five districts. The dominant genotype in the Capricorn District was LAM - 16.2%) followed by T (14.0%) and Beijing (10.9%). In Mopani district, LAM (20.2%) was the most common followed by the Beijing (18.8). The Sekhukhune district was dominated by the Beijing family (26.3%) followed by T family (5.8%). In the Vhembe district, the Beijing family was the most common (23.3%) followed by the LAM11-ZWE (13.3%). The Waterberg district had a high frequency of Beijing genotype (10.0%). Of the 215 isolates, 134 isolates with identified lineages were grouped into clusters. At Province level, 15 clusters were identified. The highest number of clusters and genotype families was observed in the Capricorn district, while Mopani district had the least number of clusters and genotype families (Fig. 3).

## Discussion

This study represents the first report on the genetic diversity and the frequency of *Mycobacterium tuberculosis* complex strains circulating in the province as well as their distribution across the five districts of the Limpopo Province. The diversity of *M. tuberculosis* strains has been described in the Western Cape, KwaZulu Natal, Gauteng, Mpumalanga, North West and Limpopo and the Beijing lineage was found to be one of the predominant strains. The present study revealed high diversity of strains, predominantly the non-Beijing lineages: LAM (LAM3, LAM9 and LAM11-ZWE), T (T1, T2, T3 and T2/X1), CAS (CAS1-Delhi and CAS1-KILI), MANU2, U, X (X1, X2, and X3), S including the Beijing and Beijing-like families. The most common lineages observed in this study were the LAM and the T, consistent with findings of studies in the KwaZulu Natal and the Eastern Cape [15–17]. The results provide an overview of the genetic profile of *M. tuberculosis* families across the districts suggesting similar genetic profile of *M. tuberculosis* families in countries bordering the Limpopo Province because of ongoing migration within the province and the neighbouring countries.

Capricorn, largest district and strategically located in the center of the Province and the neighboring country, Botswana to the North of the mining area, recorded the highest rate of *M. tuberculosis* cases with the most diverse lineages and sub-lineages T1 (7.0%), CAS-Delhi (1.0%), CAS-KILI (1.0%), LAM3 (7.4%), LAM11-ZWE (3.0%), LAM4 (1%), LAM9 (1.9%), MANU2 (2%), S (6.0%), T3 (0.5%), X3 (1.0%). Family 33 (0.5%) belonging to the Euro Amerian lineage and *M. microti* sublineage (0.9%) which is rare in humans, but may cause illness in immunocompetent patients. The Sekhukhune district recorded the second highest frequency of TB cases, with occurrence of the T1 (3.2%, T2 (2.6%) and more diverse LAM -LAM9 (0.9%), LAM3 (0.9% and LAM8 (1.4) % of the non-Beijing families and 23.7% of the Beijing strains. The Vhembe district with Zimbabwe as a neighbouring country has the third highest frequency of TB with T1 (2.3%), T2 (3.3%), LAM11-ZWE (13.3%), LAM3 (0.9%), LAM4 (10.0%), LAM8 (1.4%) and MANU2 (3.3%) of the non-Beijing strains and 23.3%0f the Beijing strains. The situation in this district that has Mozambique and Zimbabwe as neighbouring countries is consistent with earlier reports indicating LAM Spoligotype described as the predominant strain amongst Zimbabweans [10, 21–23]. Mopani district with Mozambique as a neighbouring country has *M. tuberculosis* strains of Beijing (18.8%), LAM3 (6.3%), LAM4 (12.5%), U (6.3%), X2 (0.5%). Family 33 (0.9%) was also found in this district, which also have mines in this province. The Waterberg district with Botswana as its neighbour was found to have a wide strain family diversity, predominantly the Beijing family (10,0%) followed by CAS1 DEHLI (6.7%) and *M. Microti* (0.5%).

At Province level, 134 isolates out of the 215 genotyped isolates were grouped into 15 clusters and showed a 60% clustering percentage. Majority of the isolates were clustered in the Beijing (25%), T1 (18.5%), LAM3 (11.5%) and S (9.2%) lineages. Capricorn had the highest number of clusters followed by Waterberg, Vhembe, Sekhukhune and Mopani. Within Capricorn district, the highest number of isolates clustered in the LAM3 (11.9%), while in Mopani, Sekhukhune and Vhembe districts the highest number of isolates (18.8%), (23.7%) and (23.3%), respectively were clustered in the Beijing family. Waterberg had a high number of isolates (13.3%) clustered in T1 lineage.

A limitation of this study includes the use of only the spoligotyping technique, with a low discriminatory power, and thus may have overestimated the clustering results and limited further resolution of genotypes There is an inherent limitation within the Spoligotyping international database (SpolDB4) resulting in a high percentage of orphans (37.7%) in our study similar to the results of 43% orphans (17). However, the study provides valuable and useful information for comparison with other published studies from both within and outside of South Africa. Furthermore, the study has provided information regarding the diversity of *M. tuberculosis* strains in the five districts of Limpopo Province and areas where transmission of *M. tuberculosis* is high, suggesting the need for early diagnosis and timeous treatment.

## Conclusion

The study demonstrated that the TB epidemic in the Limpopo Province is caused by a wide diversity of lineages. A high diversity of *M. tuberculosis* strains was also demonstrated at district level. The non-Beijing family was the most frequent with predominant LAM and T1 genotype lineages. A high number of clusters per district provided evidence of on-going transmission in this province. Further studies employing other techniques such as mycobacterial interspersed repetitive units-variable-number of tandem DNA repeats (MIRU-VNTR) are needed in future to confirm the current findings. There is also a need to strengthen existing tuberculosis control measures in the Province.

## Abbreviations

DNA: Deoxyribonucleic acid
HIV: Human immunodeficiency virus
MIRU-VNTR: Mycobacterial interspaced repetitive units of variable-number tandem repeats
MTB: Mycobacterium tuberculosis
RFLP: Restriction fragment length polymorphism
TB: Tuberculosis
